# Development of in-house ELISA for detection of antibodies against lumpy skin disease virus in cattle and assessment of its performance using a bayesian approach

**DOI:** 10.1016/j.heliyon.2023.e13499

**Published:** 2023-02-04

**Authors:** Nattawooti Sthitmatee, Pallop Tankaew, Wittawat Modethed, Amarin Rittipornlertrak, Anucha Muenthaisong, Nisachon Apinda, Pongpisid Koonyosying, Boondarika Nambooppha, Paweena Chomjit, Kanokwan Sangkakam, Tawatchai Singhla, Paramintra Vinitchaikul, Kittikorn Boonsri, Kidsadagon Pringproa, Veerasak Punyapornwithaya, Khwanchai Kreausukon

**Affiliations:** a Laboratory of Veterinary Vaccine and Biological Products, Faculty of Veterinary Medicine, Chiang Mai University, Chiang Mai, Thailand; b Department of Veterinary Biosciences and Public Health, Faculty of Veterinary Medicine, Chiang Mai University, Chiang Mai, Thailand; c The Veterinary Diagnostic Center, Chiang Mai University Animal Hospital, Faculty of Veterinary Medicine, Chiang Mai University, Chiang Mai, Thailand; d Herd Health Unit, The Fifth Regional Livestock Office, Department of Livestock Development, Ministry of Agriculture and Cooperative, Muang, Chiang Mai, Thailand; e Ruminant Clinic, Department of Food Animal Clinics, Faculty of Veterinary Medicine, Chiang Mai University, Chiang Mai, Thailand; f Excellent Center in Veterinary Bioscience, Chiang Mai University, Chiang Mai, Thailand; g Excellent Center for Veterinary Public Health, Chiang Mai University, Chiang Mai, Thailand

**Keywords:** Bayesian latent class analysis, Indirect ELISA, Lumpy skin disease, Sensitivity, Specificity

## Abstract

Lumpy skin disease (LSD) is a contagious disease among cattle and buffalo worldwide. Currently, an enzyme-linked immunosorbent assay (ELISA) has been recognized as an efficient diagnostic tool that is less time-consuming and easier than the viral neutralization test to measure the antibody levels. In the present study, an in-house method of indirect ELISA was developed to detect the bovine antibodies against Lumpy skin disease virus (LSDV) and its performance was assessed using field samples. This in-house method has been compared with the commercial ELISA test kit for detection of bovine antibodies against LSDV. The sensitivity (Se) and the specificity (Sp) of the test were estimated using a Bayesian latent class model. Checkerboard titration was performed using the naturally LSDV-infected bovine sera and colostrum-deprived calf sera. The LSDV antigen concentrations (1 TCID_50_/mL), the sample serum (1:500), and goat anti-bovine immunoglobulin G (IgG) labeled with horseradish peroxidase (HRP) (1:10,000) were determined to be optimal for this assay. The calculated cut-off value was 0.067, and there were no differences in the results of tests that utilized positive and negative sera (*p* < 0.05). The characteristics of two diagnostic tests were evaluated using a conditional dependent and one-population Bayesian model. The Se value of an in-house indirect ELISA were almost similar to ELISA test kit. On the other hand, the Sp value of the in-house ELISA test was lower than that of the commercial ELISA test with the median values of 89% (95% PPI = 75.9–99.3%) and 91.4% (95% PPI = 85.3–95.5%), respectively. A posterior estimate for the prevalence was 66.9% (95% PPI = 60.8–83.3%) and higher than initially expected.

## 1 Introduction

Lumpy skin disease (LSD) is an infectious disease affecting cattle and buffalo worldwide. This disease was first reported in Zambia on the African continent in 1929. The first report of LSD in Thailand was recently recorded, and it has subsequently spread throughout the country [1]. LSD is caused by the Lumpy skin disease virus (LSDV) belonging to the family *Poxviridae*, subfamily *Chordopoxvirinae*, genus *Capripoxvirus*. LSDV is a double-stranded DNA virus containing around 150 kilobase pairs (kbp) enclosed in a lipid envelope. It is relatively large (230–260 nm). In addition to LSDV, the genus *Capripoxvirus* also consists of the sheeppox virus (SPV) and the goatpox virus (GPV) exhibiting a high nucleotide identity (97%) [[2], [3], [4]]. Accordingly, the content and organization of the LSDV gene would indicate a close structural and functional relationship to other members of the subfamily chordopoxvirus of the Poxviridae family, particularly to yatapoxviruses and leporipoxviruses [2].

The World Organization for Animal Health (WOAH) suggests that the detection of antibodies against LSDV can be achieved using the virus neutralization test (VN) as a gold standard, while other alternative serological methods, including an enzyme-linked immunosorbent assay (ELISA), can be also performed [5]. The VN assay is a highly sensitive and specific test that can measure the titer of neutralizing antibodies post-infection or after a vaccination has been administered. However, the VN test not only requires technical skill and experience for the execution and interpretation, it also needs to be performed in a BSL3 laboratory. Moreover, it is more time-consuming to perform compared with the ELISA method. According to WOAH recommendations, the VN assay is suitable for use in monitoring herds and the individual status of cattle for exposure to LSDV infection. A previous study has indicated that the VN assay can be used to confirm the clinical status of infected cattle. Moreover, it can be used to establish an eradication policy, to identify the prevalence of LSD, or to determine the immune status of vaccinated cattle within the heard [6]. Importantly, the European Food Safety Authority (EFSA) scientific report on lumpy skin disease has reported that cattle with mild or asymptomatic infection will often not develop detectable antibodies with VN assay. Likewise, the vaccinated cattle will develop low levels of antibodies below the detection limit of VN assay [7].

The ELISA method is one of the serological techniques most often used for antibodies detection due to rapid, high sensitivity (Se) and specificity (Sp). It has a cheaper cost than the VN assay and can be administered more efficiently to a large number of samples on 96 well-plates [8]. This method has been successfully applied and performed in the detection of certain antibodies to animal diseases [[9], [10], [11], [12], [13]] but would require the use of a standard coated antigen in the microtiter plates [8]. Previous investigations have demonstrated that ELISA can serve as an efficient diagnostic tool for antibody detection of LSD in animals [6,14]. It was used to detect antibodies against LSD within specific regions in these studies. Nevertheless, a standard ELISA assay has not been established yet for pratical using in the first outbreak area in Thailand. Access to a reliable screening serological test kit would be crucial during a disease outbreak. Thus, it is important to develop an efficient in-house test kit with acceptable values of sensivity and specificity. The aim of this study was to develop an in-house ELISA method using a local LSDV strain as a coating antigen for the detection of antibodies against LSD in cattle. Importantly, in some situations, the true prevalence of the disease is unknown and the gold standard protocol is lacking. Therefore, Bayesian latent class analysis has been applied to address these concerns. This method has been known to use inferred posterior estimates obtained from a combination of prior information and has also applied observed data to correct the uncertainty of any unknown parameters such as test performance or disease prevalence [15]. This analysis has become increasingly used in veterinary epidemiology to evaluate the accuracy of a diagnostic test or the true disease prevalence when a gold standard is not available [15,16]. The Bayesian analysis has been employed to calculate the sensitivity and specificity of an in-house ELISA method in previous studies [9,12,13]. The Bayesian latent class model can then be performed to determine the sensitivity and specificity of the present in-house test kit when compared to the commercial test kit.

## 2 Materials and methods

### 2.1 Preparation of coating antigen for an in-house ELISA

The local LSDV strain LSD/THA/CMU/21/05 (Accession number: ON024907) used in this study was isolated from scar and skin nodules obtained from infected cattle presenting classical clinical signs of lumpy skin disease as coating antigens. The virus was isolated and cultured in MDBK cells (ATCC code CCL-34) according to the method previously described [18]. The virus titer was calculated according to the Spearman-Karber method. Subsequently, the viral particle purification was performed through a 36% w/v sucrose cushion. Briefly, the cell pellets were collected, resuspended in 10 mM Tris-HCl buffer (pH 9.0) and lysed using a sonicator 3 times for 15 s each with a 1 min rest (0.5 cycles, 80% amplitude). The suspension was centrifuged at 1040×*g* for 15 min at 4 °C to remove nuclei and other cell debris. The supernatant was saved in the new 50 ml conical tube on ice and the pellet was resuspended in 5 ml of 10 mM Tris-HCl, pH 9.0. The sonication step was repeated and centrifuged at 1930×*g* for 15 min at 4 °C. The supernatant was stored and combined with the previous collection. The supernatant containing virus was layered on 18 ml of 36% sucrose in a 40 ml Ultraclear tube (Beckman Coulter) and centrifuged at 18,000 rpm for 80 min at 4 °C in an Optima™ L-100XP Ultracentrifuge using a SW-28 rotor (Beckman Coulter). The supernatant was discarded and the pellet was resuspended in 5 ml of cold 10 mM Tris-HCl, pH 8.0. Subsequently, further purification was performed through 24–40% sucrose step gradients. From the bottom to the top, an ultracentrifuge tube was filled with 2 ml each of cold 40, 36, 32, 28, and 24% in 10 mM Tris-HCl pH 8.0, respectively. The supernatant containing the virus was loaded on top of the gradients without mixing with the top sucrose solution and centrifuged at 14,000 rpm for 40 min at 4 °C using a SW-41 rotor. The purified viral band between the 32% and 36% sucrose layers was carefully pipetted and transferred to a new ultracentrifuge tube. The purified virus was pelleted by centrifugation at 16,000 rpm in SW-41 rotor at 4 °C for 60 min. The supernatant was discarded and the pellet was resuspended in 1 ml of cold 10 mM Tris-HCl, pH 8.0. Sodium Dodecyl Sulfate polyacrylamide gel electrophoresis (SDS-PAGE) was performed to check the purity. To produce the coating antigen, purified viral particles were then inactivated by being fixed with 0.3% formalinized buffer for 24 h. They were then washed three times with PBS (pH 7.2) before dilution with PBS (pH 7.2) and kept at 4 °C until being used. In addition, the purified viral particles were confirmed by employing the PCR method as has been previously described [19].

### 2.2 Optimization of conditions for in-house indirect ELISA

The optimization of an in-house ELISA was modified from a previously described method [12]. A total of 50 sera samples were derived from infected cattle exhibiting classical cinical signs of LSDV infection and confirmed with the VN assay. They were then were employed as positive bovine sera. Subsequently, 50 colostrum-deprived neonatal calf sera were used as a VN assay-based negative LSDV serum control. Checkerboard titration involving serial dilutions of the plate-coating antigen, positive sera, and negative sera was performed under the prescribed ELISA conditions. Viral suspensions were adjusted to 10^6^ TCID_50_/ml to prepare a ten-fold dilution that would be used as a coating antigen. The reference sera ranged from 1:10 to 1:10^6^, respectively. HRP-conjugated antibody was used at dilutions of 1:1000 to 1:10,000. The optimal serum dilution and coating antigen concentration were defined as those at which the ratio was the greatest between the positive and negative sample ODs. The optimal coating concentration of the coating antigen was then used for all subsequent ELISA tests. The ELISA reaction was performed in 96-well immunoplate (Nunc-Immuno™ MaxiSorp™; Sigma-Aldrich, St. Louis, MO, USA). Each well was coated with 100 μl of the coating antigen that had been diluted in 0.05 M carbonate buffer (pH 9.6) and incubated at room temperature for 1 h. After being washed three times with washing buffer (PBST; 0.05% Tween 20 in 0.01 M PBS, pH 7.2), plates were blocked with 100 μl per well of blocking buffer (1% bovine serum albumin in 0.01 M PBS, pH 7.2) for 1 h at room temperature. After three washings with PBST, 100 μl of the serum diluted in blocking buffer was added to each well, and specimens were incubated for 1 h at room temperature. After washing the plates three more times with PBST, 100 μl of goat anti-bovine immunoglobulin G (IgG) labeled with horseradish peroxidase (HRP) (KPL, Gaithersburg, MD, USA) that had been diluted in blocking buffer was added and the plates were then incubated at room temperature for 1 h. Finally, 100 μl of 3,3’,5,5’-tetramethylbenzidine (KPL) was added to each well after another three washes and the plates were then incubated at room temperature in the dark for 15 min. The reaction was terminated by adding 50 μl of 3 M H_2_SO_4_. The optical density at a wavelength of 450 nm (OD_450_) was measured using an automatic ELISA plate reader (AccuReader; Metertech, Taipei, Taiwan).

### 2.3 Calculation of cut-off values

A number of negative LSDV serum control smples, the same set of negative sera as in the previous section, were determined to be negative for *Capripoxvirus* by immunoblotting. These samples were tested in order to determine the relevant ELISA cut-off value based on the following formula: Cut-off value = Mean OD_450_ + three standard deviations [20]. Intraplate and day-to-day variations of the ELISA were examined by repeated analysis of negative and positive sera. These were tested in 50 duplicate samples on 7 different days. The mean of each duplicate was calculated. The resulting averages were defined as standard OD values.

### 2.4 Evaluation of specificity

The specificity of the indirect ELISA was evaluated by testing sera obtained from samples derived from infected cattle displaying the classical cinical signs of LSDV infection. The tested sera are referred to as colostrum-deprived neonatal calf sera. The VN test was performed in order to confirm the positive or negative LSDV sera. In addition, the positive bovine serum obtained from cattle infected with certain other diseases including bovine viral diarrhea (BVD), foot and mouth disease (FMD), contagious bovine pleuropneumonia (CBPP) and bovine tuberculosis (bTB), had been used to determine the specificity of the ELISA method. Moreover, the VN assay was performed to validate these sera samples and all of them were identified as negative LSDV sera.

### 2.5 Sample collection

Bovine sera were kindly provided by the Lumpy Skin Disease Research Project, the Faculty of Veterinary Medicine, Chiang Mai University. A total of 460 dairy cows sera were included in this study. The 124 serum samples collected before the LSDV outbreak in Thailand were obtained from the previous study [13] as negative sera. The 336 serum samples of bias toward infected cows showing classical clinical signs such as skin nodules and were confirmed by PCR, were collected from 28 LSDV-infected herds located in four districts of Chiang Mai Province and two districts of Lamphun Province during the period of July to September 2021 as positive sera.

### 2.6 Agreement of test results of an in-house indirect ELISA test and commercial ELISA test kit

A total of 460 dairy cow sera were tested with the novel in-house ELISA test and the ID Screen® Capripox Double Antigen Multi-species test kit (ID-VET, Grabels, France). With regard to the ID Screen® Capripox Double Antigen Multi-species test kit, the testing of specific antibodies against LDSV was carried out according to the relevant instruction manual. The fundamental process for this commercial test kit included employing a double antigen ELISA by coating the ELISA plate with a capripoxvirus purified antigen. The specificity of the test involving cattle was determined to be 99.7% (CI_95%_ = 99.2–99.9%; ID-VET).

An agreement between the results of the two ELISA tests was assessed using Cohen’s kappa analysis. The agreement results were categorized into six categories based on kappa values (0–1) into slight (0–0.20), fair (0.21–0.40), moderate (0.41–0.60), substantial (0.61–0.80), and almost perfect (0.80–1.0) agreements [21].

### 2.7 Estimation of the sensitivity and specificity

Bayesian latent class modeling was conducted to estimate the sensitivity (Se) and specificity (Sp) of both the in-house and commercial ELISA tests as demonstrated by previous reports [15,17]. Since the principals of both ELISA tests are based on the detection of an antibody response, the results of the two tests were considered to be conditionally dependent [22]. Sera were obtained from farms located in the same area, wherein herd management practices were known to be similar. Therefore, these sera were assumed to have been obtained from a single population, and the Bayesian model was inferred under conditional dependence and one population model. Prior information on test performance and disease prevalence was included into the analysis using beta distributions. The Bayesian model assumed that for the k populations, the counts (Y_k_) of different combinations of the test results in terms of +/+, +/−, −/+ and −/− for the two tests followed a multinomial distribution: Y_k_ | P_qrk_ ∼ multinomial (nk, {P_qrk_}), where qr was the multinomial cell probability for the two-test outcome combinations and P_qrk_ represented a vector of probabilities for observing individual combinations of the test results [15]. Prior distributions for the commercial ELISA test were derived from published reports, whereas prior results for the in-house ELISA test and the value of prevalence of the disease were provided in the form of expert opinions as are shown in Table 1, Table 2 [6,23]. Analyses were performed in JAGS 4.3.0 via rjags and R2jags packages of R version 4.1.0 [[24], [25], [26]]. After 100,000 iterations of the model, posterior distributions were computed by discarding the first 10,000 iterations as a burn-in phase.

**Table 1:** Information of characteristics of in-house ELISA and a commercial ELISA tests for Lumpy skin disease and prevalence in Thailand.

Diagnostic Tests	Sensitivity	Specificity	Disease prevalence	References
Commercial ELISA	88.2%	99.2%		[6]
	91.0%	87.0%		[23]
In-house ELISA	N/A	N/A		N/A
Disease prevalence			40%	[1]

**Table 2:** Prior estimates for mode and 95% confidence interval (CI) for sensitivity and specificity values of in-house and commercial ELISA tests and prevalence of the disease (%).

Diagnostic Tests	Parameters	Mode	95% CI^a^
In-house ELISA^b^	Sensitivity	–	Non-informative distributions
	Specificity	–	Non-informative distributions
Commercial ELISA^c^	Sensitivity	90.0	>88.0
	Specificity	92.0	>87.0
Disease prevalence		40.0	<60.0

a 95% lower or upper credibility interval bound.

b In-house ELISA.

c Commercial ELISA.

Convergence and a lack of autocorrelation of the model were tested by visual inspection of the Gelman–Rubin diagnostic plots using three sample chains with different initial values [27,28]. Approximate convergence was indicated when the upper limit was close to 1. The degree of goodness of fit for the models was evaluated using the deviance information criterion (DIC), while the numbers of effectively estimated parameters (pD) were used as calibrating parameters [29,30]. Models with a smaller DIC were preferred to models with a larger DIC [16].

The influence of the prior information and the assumption of conditional dependence between the two ELISA tests on the posterior estimates were evaluated by model sensitivity analysis [15,16]. These analyses were performed by replacing each prior value with a non-informative uniform (0,1) distribution value and by comparing the DIC values of the models with and without the covariance term.

## 3 Results

### 3.1 Optimization of an in-house indirect ELISA

The signal-to-noise (S/N) ratio of the positive and negative sample OD_450_ indicated a concentration of the LSD coating antigen 1 TCID_50_/well, the sample serum (1:500), and HRP-conjugated goat anti-bovine IgG (1:10,000) were optimal in terms of suitable dilutions for the ELISA assay (Table 3, Table 4). The calculated cut-off value was recorded at 0.067. The OD range for the positive control sample was 0.270–0.350, while the negative control range was 0.035–0.067.

**Table 3:** The checkerboard titration of the ELISA test; the signal-to-noise (S/N) ratio was used to determine the optimal control serum dilution and antigen concentration (conjugated dilution 1:2000).

Antigen(s)	Dilution of antigen concentration	TCID_50_	S/N ratio				
			1:100	1:200	1:400	1:500	1:1000
Cmu-01	1:10	10^5^	4.259	4.259	3.186	2.536	1.584
	1:100	10^4^	5.412	5.012	2.314	1.848	1.618
	1:1000	10^3^	6.206	6.126	2.843	2.153	1.952
	1:10,000	10^2^	3.611	3.552	2.877	2.215	2.055
	1:100,000	10^1^	4.128	4.035	3.372	3.229	2.811
	1:1,000,000	1	5.319	4.941	5.854	6.717^a^	5.612

a Represents the suitable S/N ratio from this study.

**Table 4:** S/N ratios used to determine the optimal conjugated dilution and antigen concentration (control serum dilution 1:500).

Antigen(s)	Dilution of antigen concentration	TCID_50_	S/N ratio				
			1:1000	1:2000	1:4000	1:5000	1:10,000
Cmu-01	1:10	10^5^	2.534	2.223	2.198	1.169	1.037
	1:100	10^4^	2.058	2.035	2.016	1.882	2.257
	1:1000	10^3^	2.584	2.400	2.536	1.995	2.380
	1:10,000	10^2^	2.218	2.211	1.948	1.894	1.959
	1:100,000	10^1^	2.402	2.204	2.153	2.043	2.212
	1:1,000,000	1	2.455	2.318	2.615	2.877	3.126^a^

a Represents the suitable S/N ratio from this study.

### 3.2 Determination of cross-reactivity to other bovine diseases

Other bovine disease sera were tested with our present in-house indirect ELISA. The results showed the ODs of the positive sera of the other bovine diseases were lower than the calculated cut-off OD value of the present in-house ELISA (Table 5). The results indicated that our in-house indirect ELISA was not immunoreactive to the antibody against other bovine diseases.

**Table 5:** Cross-reactivity to other bovine disease positive antibodies that performed by an in-house ELISA.

Positive bovine sera	Mean ± standard deviation^a^
Foot and mouth disease (FMD)	0.049 ± 0.004
Bovine viral diarrhea (BVD)	0.053 ± 0.002
Bovine tuberculosis (bTB)	0.051 ± 0.002
Contagious bovine pleuropneumonia (CBPP)	0.045 ± 0.003
Negative control sera	0.055 ± 0.007
Positive control sera	0.363 ± 0.003

a The numbers in the table represented the average OD number and standard deviation of the bovine positive sera to diseases and the negative sera.

### 3.3 The performance of an in-house indirect ELISA test

As the results, 290 of serum samples were positive, whereas 138 of serum samples were negative to both tests. There were 22 dairy cow sera that were positive to the in-house ELISA but negative to the commercial ELISA test kit, whereas 10 dairy cow sera were negative to the in-house ELISA test and positive to the commercial ELISA test (Table 6). Notably, the agreement between the two tests was almost perfect (kappa = 0.84). Moreover, Supplementary Table 1 presents the average and standard deviation values of all bovine sera used in this study.

**Table 6:** Cross-classified test results for lumpy skin disease in 460 animals from in-house ELISA and commercial ELISA tests.

Diagnostic Test	in-house ELISA + a	in-house ELISA -b	Total
commercial ELISA +^c^	290	10	300
commercial ELISA -d	22	138	160
**Total**	**312**	**148**	**460**

a Number of positive results for the in-house ELISA test.

b Number of negative results for the in-house ELISA test.

c Number of positive results for the commercial ELISA test.

d Number of negative results for the commercial ELISA test.

### 3.4 Bayesian models to estimate the sensitivity and specificity of an in-house indirect ELISA

The median value of the posterior estimate for Se of the novel in-house ELISA test was 94.9% [95% posterior probability interval (PPI) = 86.7–99.7%)], whereas the median value of the posterior estimate for Sp of the test was 89.8% (95% PPI = 75.9–99.3%) as is shown in Table 7. The estimated Se of the commercial ELISA test was slightly higher than the prior value with a median value of 91.3% (95% PPI = 86.0–94.9%), while the estimated Sp was close to its prior value (median = 91.4, 95% PPI = 85.3–95.5%). Posterior estimates for disease prevalence were higher than the prior values with a median value of 66.9% (95% PPI = 60.8–83.3%). The conditional dependence among the two ELISA tests was low in both infected and non-infected cattle with probability intervals that included 0. The conditional independent model, which did not include a covariance term between the two tests, had a higher DIC value than the conditional dependent model (23.7 vs. 22.2, respectively); thus, the conditional dependent model was preferred as the final model.

**Table 7:** Posterior estimates for median and 95% posterior probability interval (PPI) for sensitivity and specificity of in-house ELISA and commercial ELISA tests, and prevalence of the disease (%).

Diagnostic Tests	Parameters	Median	95% PPI^a^
In-house ELISA	Sensitivity	94.9	86.7–99.7
	Specificity	89.8	75.9–99.3
Commercial ELISA	Sensitivity	91.3	86.0–94.9
	Specificity	91.4	85.3–95.5
Disease prevalence		66.9	60.8–83.3

a 95% PPI: 95% posterior probability interval.

For the purposes of convergence testing, the model exhibited proper convergence and autocorrelation was eliminated by burning out one every ten iterations. With regard to sensitivity analysis, no major changes (change in median or 95% probability percentiles >25%) were observed in the posterior estimates of the Se of the commercial ELISA test and disease prevalence when non-informative distributions were used as prior values for any of the parameters. This was interpreted as evidence of the robustness of the model. However, changes in the posterior estimates for the Sp of the commercial ELISA test were observed with a lower estimated posterior specificity (from 91.4% to 59.1%) (Supplementary Tables 2-4).

## 4 Discussion

The ELISA method has been empolyed as diagnostic tool to evaluate the immunity response against LSDV in several studies [6,14,31,32]. The majority of the outcomes of those studies have been compared with the results of the VN assay. Samojlović et al. administered the commercial ELISA test kit for detection of the antibody against LSDV from cattle sera samples in order to compare the performance of the ELISA kit with the VN assay [6]. The results obtained from the VN assay and ELISA kit were then used to calculate the kappa index. It was found that those two comparing methods were in almost complete agreement. Consequently, it was concluded that the commercial ELISA test kit, which is the same as the test kit used in our present study, exhibited the sensitivity and specificity closed to those of the VN assay. Remarkably, the VN assay is recognized as the WOAH’s recommended virus neutralization test [5]. The values of Se and Sp of the VN assay and commercial ELISA were calcaulated and the results indicated that the Se of VN was 100%, while that of the ELISA was 88.24%. The Sp of the VN assay was 100%, while the SP of the ELISA was 99.2% [6]. Moreover, Krešic′et al. compared the results of the VN assay and the commercial ELISA kit [18]. Subsequently, the Se and Sp were found to be in accordance with the results derived from Samojlović et al. [6]. The Se of VN was 94.91, while that of the ELISA was 95%. Furthermore, the Sp of the VN assay was 100%, while that of ELISA was 97.56%, respectively [18]. These results indicate that the commercial ELISA was an efficient serological tool for detection of the antibody to LSD and was actually as effective as the gold standard method. With this evidence, we have established the commercial ELISA as a standard method for comparison of results derived from our in-house ELISA. Accordingly, this diagnostic kit can be used as a gold standard method in place of the VN assay.

The use of a specific antigen is crucial for the ELISA test because of its specificity to antibody detection. Thus the search for an appropriate coating antigen is very important. Previous studies have been conducted to identify a suitable antigen to be coated onto the ELISA plate. Consequently, an inactivated sucrose gradient-purified SPPV [14], a mature virion envelope protein P32 [[31], [32], [33]], has been identified as a candidate coating antigen for ELISA. Accordingly, an inactivated whole virus particle based-ELISA has been successfully demonstrated and could be used to detect anti-capripoxvirus antibodies in sera obtained from animals that displayed clinical signs of the disease [14]. This previous study employed an inactivated Sheeppoxvirus as a coating antigen for ELISA and we have compared the results with the VN assay. The Se value of their ELISA was determined to be 96% with Sp value of 95%, while the corresponding Se of the VN assay was 96% with Sp value of 100%, respectively. The outcomes of a previous study support our findings, wherein an inactivated viral particle was used as a coating antigen for ELISA. Importantly, the use of an ELISA might be more convenient and suitable for local laboratories as an LSD diagnostic tool. However, our present Se (95.4%) and Sp (81.4%) values were determined to be lower than those of the previous study. It would be advantageous to improve the Se value of our in-house ELISA in future investigations. This would be important because the present study employed the field isolate that had been identified as a local strain of Capripoxvirus. Consequently, this difference might have affected the Se and Sp values of the ELISA. A previous genomic study of LSDV indicated that the LSDV was closely related to other members of the Chordopoxvirinae rather than members in the same genus. The data would also suggest that the findings indicate that this group of genes was responsible for viral host range and virulence, as well as immunogenicity [2,34]. However, certain candidate immunogenic proteins have been recommended for the ELISA, namely the p32 protein of Capripoxvirus [[31], [32], [33], [34]]. Unfortunately, certain problems have been associated with the preparation of the p32 protein and were particular to the recombinant p32 protein. Therefore, this protein has not been suggested for using in local laboratoties or to be employed as a routine coating antigen for their in-house ELISA. According to our findings, the local strain of LSDV that was used as a coating antigen in the present study produced efficient values of Se and Sp when compared to the commercial ELISA kit. The evidence of our findings prove that the local strain can be used as an efficient coating antigen for an in-house ELISA. This outcome will assist local loboratories in establishing their own diagnostic tools for the monitoring of the antibody to LSDV. In addition, an almost perfect agreement was observed between both ELISA tests using Cohen’s kappa analysis. The high correlation between the test outcomes suggests that their application as serial tests would help to increase the overall specificity or the performance of LSD testing [35].

A Bayesian latent class analysis has been performed for the estimation of accuracy of a diagnostic test and has been used to evaluate the values of Se and Sp of various diagnostic techniques in veterinary medicine, especially with regard to the development of a novel diagnostic test [10,11,13,15]. The posterior estimates for the Se of the commercial ELISA test were similar to the results of a previous study conducted in Serbia and slightly higher than the findings of yet another study conducted in Serbia. The studies had reported the Se values of 91.0% and 88.2%, respectively [6,23]. Furthermore, the estimated Sp value of the commercial test was lower than that of the previous study conducted in Sebia and slightly higher than the other Serbian study with Sp values of 99.2% and 87.0%, respectively [6,23]. Nevertheless, both studies have been perfomed on vaccinated animals for the detection of anibodies against LSD. The test accuracy had been evaluated by comparing the test results with those of the VN assay. Conversely, the present study was conducted on infected animals and used a Bayesian approach for determination of the test performance. Importantly, the limitation of this study was a lack of a study or information of the commercial ELISA test characteristics resulting in less information for the prior esimates and narrow priors of both Se and Sp of the test. This may have had an impact on the model convergence and posterior estimates for the final model.

The posterior estimates for disease prevalence were higher than the prior values, which had been inferred using the outcomes of a single study conducted in the northeastern part of Thailand in conjunction with the opinions of an expert. This outcome might be explained by the fact that all samples were collected from infected herds and were considered to be high prevalence samples. Moreover, the results might suggest that the prevalence of the LSD in this region was higher than the northeastern region by what had initially been expected. With regard to the model sensitivity analysis, there were no major changes in the posterior estimates when non-informative prior distributions were applied in the model with the exception of the posterior estimates for the Sp of the commercial ELISA test. This would suggest that the prior values of this parameter did have a stronger influence on the results of the model. Thus, there may have been a strong effect of the prior selections on the posterior estimates, and further studies with larger populations ideally including more prior information are needed in order to verify the results from this study.

## 5 Conclusion

An in-house ELISA method has been successfully developed in this study. Our method used a local strain of an inactivated whole LSDV particle as a coating antigen at 1 TCID_50_/well, a sample serum at a dilution of 1:500, and HRP-conjugated goat anti-bovine IgG at a dilution of 1:10,000. The calculated cut-off value was 0.067. The posterior estimates for the Se and Sp values of an in-house ELISA (Se = 94.9%, Sp = 89.8%) developed in this study were notably higher than the Se value of the commercial ELISA kit. However, the Sp value was lower than the commercial ELISA kit of the commercial ELISA kit (Se = 91.3%, Sp = 91.4%). From these results, we can conclude that our in-house ELISA produced higher sensitivity levels along with decreases in specificity levels than those of the commercial test kit. Furthermore, the commercial ELISA kit in conjunction with the method described herein, will assist local laboratories in establishing an in-house diagnostic tool for the monitoring of an antibody to LSDV.

## Declarations

### Ethical statement

This study was conducted according to the guidelines of and approved by the Animal Care and Use Committee (FVM-ACUC) approval number S26/2563, Faculty of Veterinary Medicine, Chiang Mai University and Chiang Mai University Institutional Biosafety Committee (CMU-IBC) approval number CMUIBC A-0764012.

## Author contribution statement

Nattawooti Sthitmatee, DVM,MS,PhD: Conceived and designed the experiments; Analyzed and interpreted the data; Contributed reagents, materials, analysis tools or data; Wrote the paper.

Pallop Tankaew, M.T.,M.S: Performed the experiments; Contributed reagents, materials, analysis tools or data.

Wittawat Modethed, DVM; Paramintra Vinitchaikul, DVM,MS,PhD: Conceived and designed the experiments; Performed the experiments.

Amarin Rittipornlertrak, DVM,PhD; Anucha Muenthaisong, BS.,MS; Nisachon Apinda, DVM; Pongpisid Koonyosying, BS.,MS; Boondarika Nambooppha, DVM,PhD; Paweena Chomjit, BS; Kanokwan Sangkakam, BS: Performed the experiments.

Tawatchai Singhla, DVM,MS,PhD: Conceived and designed the experiments; Performed the experiments; Analyzed and interpreted the data; Contributed reagents, materials, analysis tools or data; Wrote the paper.

Kittikorn Boonsri, DVM.,MS; Kidsadagon Pringproa, DVM,MS,PhD: Conceived and designed the experiments.

Verrasak Punyapornwithaya, DVM,MS,PhD; Khwanchai Kreausukon, DVM,MS,Dr.med.vet: Conceived and designed the experiments; Analyzed and interpreted the data.

## Funding statement

This work was supported by the National Research Council of Thailand (NRCT) [Grant No: FF66/021], Chiang Mai University [R000029530] and Faculty of Veterinary Medicine, Chiang Mai University [08/2566].

## Data availability statement

No data was used for the research described in the article.

## Declaration of interest’s statement

The authors declare no competing interests.

## References

[1] Arjkumpa O. (2021). First emergence of lumpy skin disease in cattle in Thailand, 2021. Transbound. Emerg. Dis..

[2] Tulman E.R., Afonso C.L., Lu Z., Zsak L., Kutish G.F., Rock D.L. (2001). Genome of Lumpy skin disease virus. J. Virol..

[3] Bhanuprakash V., Indrani B.K., Hosamani M., Singh R.K. (2006). The current status of sheep pox disease. Comp. Immunol. Microbiol. Infect. Dis..

[4] Hamdi J., Munyanduki H., Omari Tadlaoui K., El Harrak M., Fassi Fihri O. (2021). Capripoxvirus infections in ruminants: a review. Microorganisms.

[5] WOAH Terrestrial Manual 2021. Chapter 3.4.12. Lumpy Skin Disease. Available online: https://www.oie.int/fileadmin/Home/eng/Health_standards/tahm/3.04.12_LSD.pdf (accessed on 9 August 2021).

[6] Samojlović M. (2019). Detection of antibodies against Lumpy skin disease virus by Virus neutralization test and ELISA methods. Acta Vet..

[7] EFSA (European Food Safety Authority) (2017). Scientific report on lumpy skin disease: I. Data collection and analysis. EFSA J..

[8] Crowther J.R. (1995).

[9] Poolperm P., Varinrak T., Kataoka Y., Tragoolpua K., Sawada T., Sthitmatee N. (2017). Development and standardization of an in-house indirect ELISA for detection of duck antibody to fowl cholera. J. Microbiol. Methods.

[10] Tankaew P. (2017). Evaluation of an In-house indirect ELISA for detection of antibody against haemorrhagic septicemia in Asian elephants. J. Microbiol. Methods.

[11] Tankaew P. (2018). Comparison of two indi-rect ELISA coating antigens for the detection of dairy cow antibodies against Pasteurella multocida. J. Microbiol. Methods.

[12] Areewong C. (2020). Evaluation of an in-house indirect enzyme-linked immunosorbent assay of feline panleukopenia VP2 subunit antigen in comparison to hemagglutination inhibition assay to monitor tiger antibody levels by Bayesian ap-proach. BMC Vet. Res..

[13] Singhla T., Tankaew P., Sthitmatee N. (2020). Validation of a novel ELISA for the diagnosis of Hemorrhagic septicemia in dairy cattle from Thailand using a Bayesian approach. Vet. Sci..

[14] Babiuk S. (2009). Detection of antibodies against capripoxviruses using an inactivated sheeppox virus ELISA. Transbound. Emerg. Dis..

[15] Branscum A.J., Gardner I.A., Johnson W.O. (2005). Estimation of diagnostic-test sensitivity and specificity through Bayesian modeling. Prev. Vet. Med..

[16] Rahman A.K. (2013). Bayesian estimation of true prevalence, sensitivity and specificity of indirect ELISA, Rose Bengal Test and Slow Agglu-tination Test for the diagnosis of brucellosis in sheep and goats in Bangladesh. Pre. Vet. Med..

[17] Gardner I.A. (2002). The utility of Bayes’ theorem and Bayesian inference in veterinary clinical practice and research. Aust. Vet. J..

[18] Krešic′ N., Šimic′ I., Bedekovic′ T., Acinger-Rogic′ Ž., Lojkic′ I. (2020). Evaluation of serological tests for detection of anti-bodies against Lumpy skin disease virus. J. Clin. Microbiol..

[19] Ireland D.C., Binepal Y.S. (1998). Improved detection of capripoxvirus in biopsy samples by PCR. J. Virol. Methods.

[20] Kich J.D. (2007). Development and ap-plication of an enzyme-linked immunosorbent assay to detect antibodies against prevalent Salmonella serovars in swine in southern Brazil. J. Vet. Diagn. Invest..

[21] Landis J.R., Koch G.G. (1977). The measurement of observer agreement for categorical data. Biometrics.

[22] Gardner I.A., Stryhn H., Lind P., Collins M.T. (2000). Conditional dependence between tests affects the diagnosis and surveil-lance of animal diseases. Prev. Vet. Med..

[23] Milovanović M. (2019). Humoral immune response to repeated lumpy skin disease virus vaccination and performance of serological tests. BMC Vet. Res..

[24] Plummer M., Stukalov A., Denwood M. (2019). https://cran.r-project.org/web/packages/rjags/rjags.pdf.

[25] R. Core Team (2021). http://www.R-project.org/.

[26] Su Y.S., Yajima M. (2021). https://cran.r-project.org/web/packages/R2jags/R2jags.pdf.

[27] Gelman A., Rubin D.B. (1992). Inference from iterative simulation using multiple sequences. Stat. Sci..

[28] Brooks S.P., Gelman A. (1997). General methods for monitoring convergence of iterative simulations. J. Comput. Graph Stat..

[29] Spiegelhalter D.J., Best N.G., Carlin B.R., Van der Linde A. (2002). Bayesian measures of model complexity and fit. J. Roy. Stat. Soc..

[30] Berkvens D., Speybroek N., Praet N., Adel A., Lesaffre E. (2006). Estimating disease prevalence in a Bayesian framework us-ing probabilistic constraints. Epidemiology.

[31] Venkatesan G. (2018). Expression and evaluation of recombinant P32 protein based ELISA for sero-diagnostic potential of capri-pox in sheep and goats. Mol. Cell. Probes.

[32] Tian H., Chen Y., Wu J., Shang Y., Liu X. (2010). Serodiagnosis of sheeppox and goatpox using an indirect ELISA based on synthetic peptide targeting for the major antigen P32. Virol. J..

[33] Carn V.M., Kitching R.P., Hammond J.M., Chand P. (1994). Use of a recombinant antigen in an indirect ELISA for detecting bovine antibody to capripoxvirus. J. Virol. Methods.

[34] Kushwaha A., Kumar A., Madhavan A., Goswami D., Poulinlu G., Venkatesan G. (2019). Immunogenic proteins of capripox virus: potential applications in diagnostic/prophylactic developments. Hosts Viruses.

[35] de la Rua-Domenech R., Goodchild A.T., Vordermeier H.M., Hewinson R.G., Christiansen K.H., Clifton-Hadley R.S. (2006). Ante mortem diagnosis of tuberculosis in cattle: a review of the tuberculin tests, gamma-interferon assay and other ancillary diagnostic techniques. Res. Vet. Sci..

