# Supplementary material for: Development of in-house ELISA for detection of antibodies against lumpy skin disease virus in cattle and assessment of its performance using a bayesian approach
Source: Heliyon. 2023 Feb 4;9(2):e13499. doi: 10.1016/j.heliyon.2023.e13499 (PMC9941990; doi:10.1016/j.heliyon.2023.e13499)
Supplement: Multimedia component 1 [file mmc1.docx]

**Supplementary Table 1**

The optical density values of bovine sera used in this study

| **Bovine sera** | **in house ELISA** | | **ELISA test kit** | |
| --- | --- | --- | --- | --- |
|  | **Mean** | **SD** ^a^ | **Mean** | **SD** ^a^ |
| Positive sera to Foot and mouth disease | 0.049 | 0.004 | 0.053 | 0.005 |
| Positive sera to Bovine viral diarrhea | 0.053 | 0.002 | 0.053 | 0.002 |
| Positive sera to Bovine tuberculosis | 0.051 | 0.002 | 0.056 | 0.008 |
| Positive sera to Contagious bovine pleuropneumonia | 0.045 | 0.003 | 0.058 | 0.004 |
| Negative control sera | 0.055 | 0.007 | 0.055 | 0.004 |
| Positive control sera | 0.363 | 0.003 | 0.405 | 0.007 |
| Test sera - positive LSDV sera | 0.584 | 0.423 | 0.377 | 0.513 |
| Test sera - negative LSDV sera | 0.113 | 0.054 | 0.062 | 0.005 |

^a^ Standard deviation

**Supplementary Table 2**

Posterior estimates for characteristics of in-house ELISA and commercial ELISA tests, prevalence of the disease (%), and % changing of the median after applying of non-informative of the commercial ELISA test sensitivity.

| **Diagnostic Tests** | **Parameters** | **Median** | **95% PPI ^a^** | **% Median change** |
| --- | --- | --- | --- | --- |
| In-house ELISA | Sensitivity | 96.5 | 86.3-99.8 | 1.7 |
|  | Specificity | 86.3 | 75.3- 98.5 | 3.9 |
| Commercial ELISA | Sensitivity | 94.5 | 84.7- 99.7 | 3.5 |
|  | Specificity | 91.4 | 85.4- 95.5 | 0 |
| Disease prevalence |  | 64.8 | 58.0-73.3 | 3.1 |

^a^ 95% PPI: 95% posterior probability interval

**Supplementary Table 3**

Posterior estimates for characteristics of in-house ELISA and commercial ELISA tests, prevalence of the disease (%), and % changing of the median after applying of non-informative of the commercial ELISA test specificity.

| **Diagnostic Tests** | **Parameters** | **Median** | **95% PPI ^a^** | **% Median change** |
| --- | --- | --- | --- | --- |
| In-house ELISA | Sensitivity | 93.9 | 86.8-99.6 | 1.1 |
|  | Specificity | 57.4 | 38.2-94.6 | **36.1** |
| Commercial ELISA | Sensitivity | 90.1 | 84.2-94.4 | 1.3 |
|  | Specificity | 59.1 | 40.9- 95.0 | **35.3** |
| Disease prevalence |  | 48.7 | 23.9 -69.9 | **27.2** |

^a^ 95% PPI: 95% posterior probability interval

**Supplementary Table 4**

Posterior estimates for characteristics of in-house ELISA and commercial ELISA tests, prevalence of the disease (%), and % changing of the median after applying of non-informative of the disease prevalence.

| **Diagnostic Tests** | **Parameters** | **Median** | **95% PPI ^a^** | **% Median change** |
| --- | --- | --- | --- | --- |
| In-house ELISA | Sensitivity | 93.4 | 84.5-99.5 | 1.6 |
|  | Specificity | 90.4 | 75.5-99.4 | 0.7 |
| Commercial ELISA | Sensitivity | 90.3 | 84.0-94.4 | 1.1 |
|  | Specificity | 91.8 | 86.2-95.8 | 0.4 |
| Disease prevalence |  | 69.3 | 62.8-77.1 | 3.6 |

^a^ 95% PPI: 95% posterior probability interval
